# Ionic Liquids in Sustainable Biocatalytic Lactone Synthesis: Green Chemistry Metrics and Process Evaluation

**DOI:** 10.3390/molecules31132226

**Published:** 2026-06-24

**Authors:** Anna Wolny, Anita Procek, Igor Biały, Izabela Ziębińska, Laura Kudzia, Emilia Gielarowska

**Affiliations:** Department of Chemical Organic Technology and Petrochemistry, Faculty of Chemistry, Silesian University of Technology, Krzywoustego 4, 44-100 Gliwice, Poland; ap301258@student.polsl.pl (A.P.); ib304900@student.polsl.pl (I.B.); iz314765@student.polsl.pl (I.Z.); lk309888@student.polsl.pl (L.K.); eg309727@student.polsl.pl (E.G.)

**Keywords:** ionic liquids, biocatalysis, Baeyer–Villiger oxidation, lactones, green chemistry

## Abstract

Ionic liquids remain attractive alternatives as multifunctional media for the sustainable biosynthesis of lactones. Their unique physicochemical properties, including negligible vapor pressure, high thermal stability, and tunable polarity, offer significant advantages in terms of biocatalyst stabilization and reaction selectivity. For lactone synthesis, ionic liquids facilitate improved control over enzymatic transformations, enable efficient catalyst recycling, and reduce solvent consumption. This review summarizes recent advances in the application of ionic liquids as solvents or support modifiers in enzymatic lactone synthesis, focusing also on ε-caprolactone biosynthesis. A green chemistry metrics evaluation was also performed for selected examples from the literature. The role of ionic liquids in enhancing process efficiency and supporting green, sustainable process design is critically discussed, highlighting their potential for the development of more sustainable and environmentally friendly lactone production technologies.

## 1. Introduction

Lactones are an important class of cyclic esters that are widely used as intermediates and building blocks in the chemical, pharmaceutical, cosmetic, and polymer industries. Their versatile reactivity and tunable physicochemical properties make lactones key precursors for the production of biodegradable fine chemicals. The global demand for lactones has grown significantly over the recent decades, mainly due to their increasing use in the production of advanced materials [1]. Among lactones, ε-caprolactone is considered the most industrially important. The ε-caprolactone market, valued at $412 million in 2024, is expected to reach $738 million by 2033, highlighting its central role in industrial-scale lactone production [2]. It is a crucial monomer for the production of poly(ε-caprolactone), a biodegradable and biocompatible polyester extensively used in medical devices, drug delivery systems, tissue engineering, and packaging materials [3]. The industrial production of ε-caprolactone by major companies such as BASF, Ingevity Corporation, and Daicel Corporation is based on the Baeyer–Villiger oxidation of cyclohexanone using peracids, which are generated either in situ or in separate reactors [4]. Although these processes are well established and efficient, they are often associated with safety concerns, limited selectivity, waste generation, and the use of volatile organic solvents.

In response to increasing environmental regulations and the principles of green chemistry, researchers have been working on developing more sustainable methods for producing lactones [5,6]. The use of ionic liquids (ILs) as sustainable catalysts or reaction media is one of the “green” approaches studied for lactone synthesis [4,7,8,9]. ILs are salts composed of organic cations paired with organic or inorganic anions that remain liquid at relatively low temperatures. Their negligible vapor pressure, non-flammable nature, and high thermal stability make them promising alternatives to conventional organic solvents or catalysts in lactone synthesis [10]. For example, 1-butyl-3-methylimidazolium bis(trifluoromethanesulfonyl)imide ([bmim][NTf_2_]) was used as a solvent in the oxidation of cyclic ketones, such as cyclopentanone, cyclohexanone, 2-methylcyclohexanone, 2-norbornone, 2-adamantanone, and cycloheptanone, to the corresponding lactones at 90 °C in the presence of a free radical initiator, SnCl_4_ as a catalyst, and an oxygen/benzaldehyde system as the oxidant. This system achieved high lactone yields (84–90%) and enabled effective recycling of the ionic liquid [11]. In other studies, 1-butyl-3-methylimidazolium trifluoromethanesulfonate ([bmim][OTf]) served as a catalyst and solvent for efficient lactone synthesis at room temperature or 40 °C, in the presence of bis(trimethylsilyl) peroxide as the oxidant. High product yields (78–99%) were obtained, while the IL was successfully recycled for at least five consecutive cycles without a noticeable decrease in catalytic efficiency during the model oxidation of cyclopentanone [12].

Biocatalysis is an environmentally friendly strategy for the synthesis of lactones and many other fine chemicals, enabling high chemo-, regio-, and stereoselectivity under mild conditions. It minimizes the use of hazardous reagents and harsh reaction conditions, reducing environmental impact and supporting green chemistry practices. Chemo-enzymatic approaches for lactone production, combining chemical oxidation steps with enzymatic catalysis, have become another promising and environmentally sustainable alternative, which will be discussed in more detail in this paper. However, industrial implementation of enzymatic processes is often limited by challenges related to catalyst stability, difficulties in catalyst separation, solvent compatibility, and the efficient integration of chemical and enzymatic steps [13,14,15,16,17,18,19,20].

**Figure 1:**
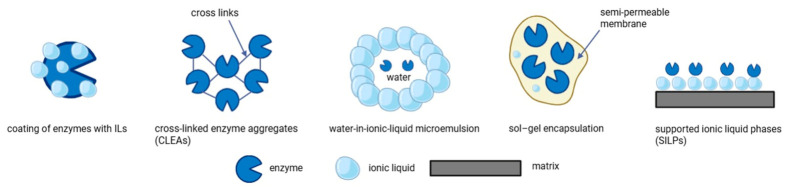
Stabilization of enzymes in ILs.

The limited stability of enzymatic catalysts remains a key challenge, and many methods for their stabilization have already been developed. ILs have been extensively studied for enzyme stabilization due to their negligible volatility and tunable physicochemical properties. The possibility of tailoring the structure of ILs through the selection of the appropriate cations and anions enables control over polarity, hydrophobicity, viscosity, and hydrogen-bonding ability, which are crucial factors for maintaining enzyme activity and stability [21,22,23]. ILs have been successfully applied in a wide range of biocatalytic transformations. Lipases are among the most extensively studied enzymes in these systems and have been used in esterification, transesterification, hydrolysis, condensation, and kinetic resolution reactions, often showing improved activity, stability, and enantioselectivity in ILs. In addition, other enzyme classes, such as oxidases, laccases, peroxidases, cytochrome P450 enzymes, proteases, and cellulases, have been reported to function efficiently in ILs. In many cases, ILs enhance the stability of the enzyme, improve its catalytic performance, selectivity, and increase substrate solubility, which is crucial for poorly soluble organic compounds [23,24]. Enzyme stabilization in ILs can be classified into two main approaches: enzyme immobilization and environment modification, illustrated in Figure 1. The first approach includes immobilization techniques such as sol–gel encapsulation [25], cross-linked enzyme aggregates (CLEAs) [26], and supported ionic liquid phases (SILPs) [23], where ILs are used as additives or as components of the support. The second method involves modification of the reaction environment, including water-in-IL microemulsions [27] and coating of enzymes with ILs [28], which protect protein conformation and improve enzyme catalytic performance. In biocatalysis, the rational design of ILs tailored to the protein and reaction system is crucial for achieving efficient enzymatic production.

In this paper, the biosynthesis of ε-caprolactone and the enzymatic production of lactones in the presence of ionic liquids will be discussed. Previously, Zhu et al. [13] reported advances in lipase-catalyzed lactone formation and ring-opening polymerization. Kroutil et al. [14] reviewed biocatalytic linear cascade reactions for lactone preparation. Subsequently, Wang et al. [15] summarized enzymatic approaches to the synthesis of lactones and lactams. After that, Fraaije et al. [16] discussed Baeyer–Villiger monooxygenases for lactone production, while Hu et al. [17] highlighted progress in lipase-catalyzed lactone synthesis, and Paul et al. [18] presented alcohol dehydrogenase-driven enzymatic cascades for lactone formation. In addition, Domingues et al. [19] critically reviewed microbial routes for lactone biosynthesis, and Irfan et al. [20] outlined enzymatic Baeyer–Villiger oxidation as a tool for lactone synthesis. This work aims to present recent advances (2000–2025) in the enzymatic synthesis of ε-caprolactone, an important monomer for biodegradable polymer production, and to discuss the role of ionic liquids as green solvents or components of supports in enzymatic lactone synthesis.

## 2. Biosynthesis of ε-Caprolactone and Its Derivatives

ε-Caprolactone is an important building block in the fine chemical and polymer industries, mainly used to produce biodegradable polyesters, polyurethanes, and functional materials. Conventional industrial synthesis of ε-caprolactone is based on chemical Baeyer–Villiger oxidation using peracids, which has led to increased recent attention to biocatalytic approaches that offer improved selectivity, milder reaction conditions, and enhanced sustainability. In particular, strategies based on enzymes and whole cells have become attractive alternatives for the green production of ε-caprolactone and its structurally related derivatives. In Table 1, selected biosynthetic approaches for the enzymatic and chemo-enzymatic production of ε-caprolactone and its alkyl derivatives are presented.

**Scheme 1:**
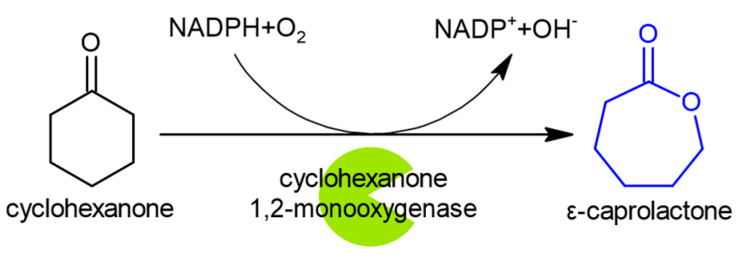
Baeyer–Villiger oxidation of cyclohexanone catalyzed by cyclohexanone monooxygenase.

The first six examples (Entries 1–6) describe the synthesis of ε-caprolactone using whole cells expressing cyclohexanone monooxygenases, in which the biocatalysts were produced by fermentation and then applied in whole-cell biotransformations. Scheme 1 illustrates the general concept of Baeyer–Villiger oxidation catalyzed by cyclohexanone monooxygenase. The enzymatic mechanism is similar to the classical chemical Baeyer–Villiger oxidation and involves activation of molecular oxygen by the reduced FAD cofactor, formation of a peroxyflavin intermediate, nucleophilic attack on the carbonyl group, and rearrangement leading to lactone formation. The Baeyer–Villiger reaction mechanism of monooxygenases was described in detail by Fraaije et al. [16] in their review.

**Scheme 2:**
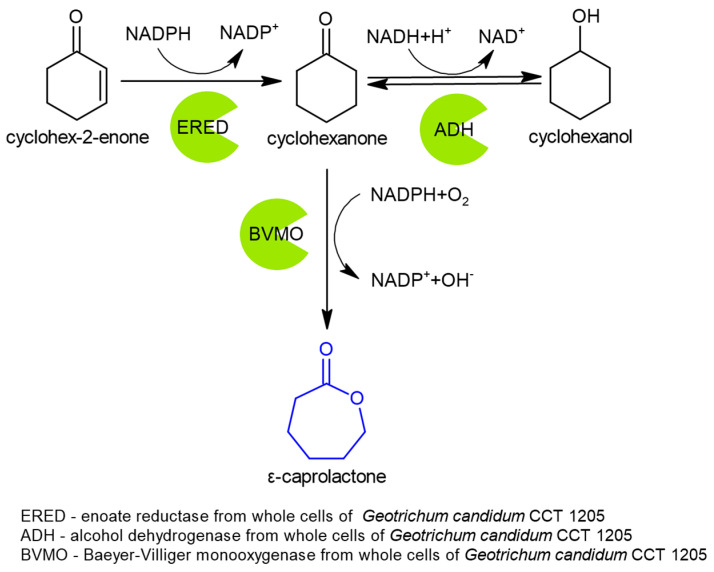
One-pot cascade biotransformations of cyclohexenone, cyclohexanol and cyclohexanone into ε-caprolactone.

Non-growing and growing engineered *E. coli* cells overexpressing cyclohexanone monooxygenase among the microorganisms used for enzymatic Baeyer–Villiger oxidation of cyclohexanone. For growing cells, ε-caprolactone production achieved 11.0 g L^−1^ in a glucose-limited fed-batch culture. Coexpression of glucose-6-phosphate dehydrogenase for NADPH regeneration increased the yield to 15.3 g L^−1^, highlighting the importance of cofactor supply in whole-cell Baeyer–Villiger oxidations [30]. On the other hand, it has been reported that non-growing cells exhibited higher productivity (0.79 g h^−1^ L^−1^) than growing cells (0.049 g h^−1^ L^−1^) in the absence of external cofactor addition, with 88% product yield. The use of glucose for in situ NADPH regeneration eliminated the need for external cofactors and resulted in a 20-fold higher volumetric productivity compared with growing cells [29]. These approaches demonstrate significant potential for scaling up whole-cell-based Baeyer–Villiger oxidation of cyclohexanone through minor process modifications. Another reported strategy for enhancing the efficiency of ε-caprolactone synthesis involved changing the microbial host from the commonly used *Escherichia coli* to *Corynebacterium glutamicum*. Recombinant *Corynebacterium glutamicum* (growing cells) expressing the chnB gene from *Acinetobacter calcoaceticus* efficiently catalyzed the Baeyer–Villiger oxidation of cyclohexanone to ε-caprolactone. Optimization of gene expression, induction, and fed-batch cultivation enabled the production of 16.0 g L^−1^ ε-caprolactone, achieving a volumetric productivity of 2.3 g L^−1^ h^−1^ (Y = 94%). These values correspond to a 2.7-fold improvement compared with recombinant *E. coli* expressing the same gene, indicating that *C. glutamicum* is a promising host for efficient Baeyer–Villiger biosynthesis [31]. Further research (Entry 4, 5) identified another microorganism, *Geotrichum candidum,* for ε-caprolactone production. In the first study, various biocatalyst forms, including growing, resting, lyophilized, and immobilized cells on different matrices, were evaluated to enhance monooxygenation efficiency. The best results were achieved with immobilization on polyacrylamide or Pterocladia agar supports, which enabled effective control over oxygen availability and selective promotion of the Baeyer–Villiger oxidation. It was also reported that under high oxygenation conditions, nearly 100% conversion to ε-caprolactone was achieved without detectable cyclohexanol formation, whereas low oxygenation conditions favored ketone reduction to cyclohexanol [32]. This work demonstrated that selective control of reaction pathways through cell immobilization or oxygen supply can effectively enhance lactone production. The use of whole cells of *Geotrichum candidum* (CCT1205) also enabled efficient production of ε-caprolactone from cyclohexenone, cyclohexanone, and cyclohexanol (Entry 5, 23, 28). The authors reported that one-pot cascade biotransformations revealed sequential reductions in the C=C and C=O bonds by ene-reductase (ERED) and alcohol dehydrogenase (ADH), followed by Baeyer–Villiger oxidation catalyzed by monooxygenase (Scheme 2). Complete conversion to the main product was observed for all three substrates with reaction times of 4–6 h. Using cyclohexanone as the starting material, the whole cells could be recycled over six consecutive cycles with minimal loss of activity (88% yield in the last cycle) [33]. This approach highlights the advantage of whole cells over genetically modified biocatalysts, providing a simpler process and eliminating the need for expensive synthetic cofactors. These results represent one of the most efficient strategies, in terms of reaction simplicity, time, ε-caprolactone yield, and scalability, based on whole cells. A very innovative strategy presented in Entry 6 was photosynthetic whole-cell biocatalysis using *Chlamydomonas reinhardtii* expressing cyclohexanone monooxygenase. In this system, conversion of cyclohexanone to ε-caprolactone was enabled through light-driven generation of O_2_ and NADPH. Optimal lactone formation (Y = 81%) was achieved under low light intensity (26 μmol photons m^−2^s^−1^) and low ethanol concentration (1.7%, *v*:*v*), thereby decreasing in the activity of competing alcohol dehydrogenase and the rate of cyclohexanol synthesis. Moreover, ε-caprolactone production was further enhanced by directing the cyclohexanone monooxygenase to the chloroplast enriched with NADPH and O_2_ [34]. The described work showed the significant potential of engineered eukaryotic photosynthetic microorganisms for industrial implementation in photobiotransformation processes.

**Scheme 3:**
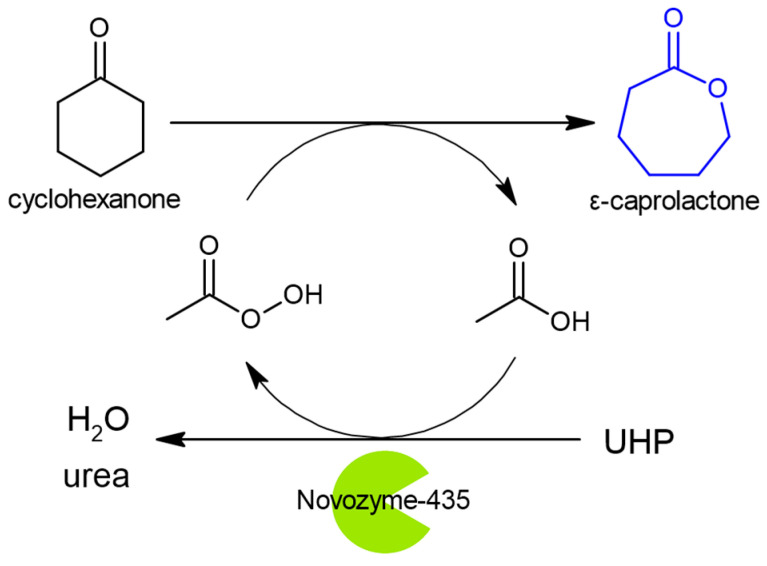
Lipase-catalyzed chemo-enzymatic Baeyer–Villiger oxidation of cyclohexanone proceeding through in situ peracid formation from carboxylic acid.

**Scheme 4:**
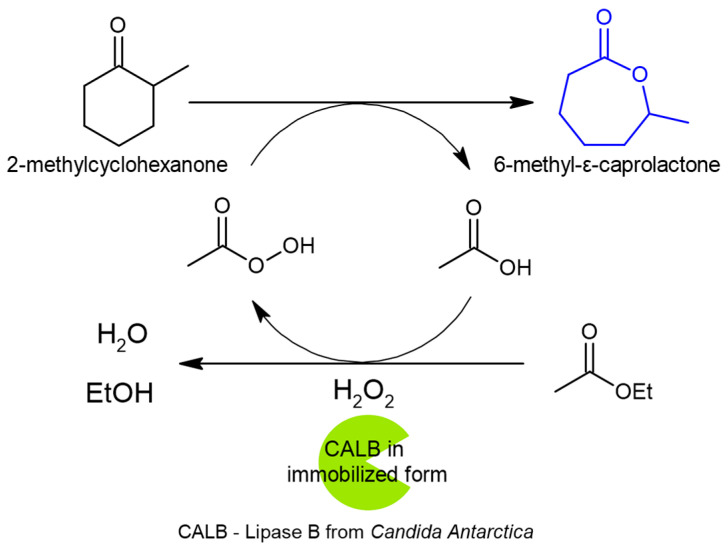
Lipase-catalyzed chemo-enzymatic Baeyer–Villiger oxidation of 2-methylcyclohexanone proceeding through in situ peracid formation from carboxylic acid ester.

A complementary approach to ε-caprolactone production and its alkyl derivatives is based on lipase-catalyzed chemo-enzymatic Baeyer–Villiger oxidation, which has attracted considerable attention for its operational simplicity and the use of non-aqueous solvents (Entry 7–22). In the presence of hydrogen peroxide or urea hydrogen peroxide (UHP), these enzymes are able to convert carboxylic acids or their esters into the corresponding peracids *via* acyl–enzyme intermediates. The in situ-generated peracids subsequently act as oxidants in Baeyer–Villiger oxidation of cyclic ketones, yielding the corresponding lactones (Scheme 3 and Scheme 4). The carboxylic acids or their esters serve a dual function: they act as precursors for peracid formation and, in the case of esters, also as reaction media. Common oxidants applied in these systems include aqueous hydrogen peroxide or UHP, which allow controlled peracid generation and limit non-selective oxidation. Compared with aqueous hydrogen peroxide, UHP is a more convenient oxidant because it is a stable solid that is easy to handle and store. Its controlled release provides a steady supply of hydrogen peroxide and avoids high local oxidant concentrations that may affect the stability of the enzyme. Compared with monooxygenase-catalyzed processes, lipase-mediated chemo-enzymatic Baeyer–Villiger oxidations do not require cofactors or regeneration systems. Lipases are commercially available and widely used in the chemical industry. The most common type is lipase B from *Candida antarctica* (CALB) in native and immobilized forms, known as Novozyme 435. Moreover, the high activity and stability of lipases under non-aqueous conditions make this approach attractive for simplified process design and for improving the solubility of a broad range of substrates.

As an example of lipase-based chemo-enzymatic Baeyer–Villiger oxidation discussed above, a system based on Novozyme 435 and perhydrolysis of ethyl acetate in the presence of UHP was reported (Entry 7, 13). Under mild conditions (cyclohexanone: UHP molar ratio 1:1.1, 1.5 mL ethyl acetate, room temperature), ε-caprolactone was obtained with approximately 80% yield after six days. Substituted cyclohexanones showed higher efficiency: 2-methylcyclohexanone afforded the corresponding ε-caprolactone in up to 95% yield. The absence of water prevented lactone hydrolysis, and only low enzyme loadings were required to achieve effective oxidation [35]. In another study, CALB immobilized as CLEAs was used for the Baeyer–Villiger oxidation of cyclohexanone to ε-caprolactone, giving 79% yield after 48 h at 40 °C (cyclohexanone: H_2_O_2_ = 1:2, ethyl acetate) (Entry 8, 14, 19). Substituted cyclohexanones showed position-dependent yields (2-methylcyclohexanone 84%, 4-methylcyclohexanone 77%). It was reported that hydrogen peroxide and acetic acid reduced enzyme stability, demonstrating the influence of substrate structure and reaction conditions on biocatalyst efficiency [36]. The next report presented CALB immobilized on silica supports with multimodal pores or modified SBA-15 applied for Baeyer–Villiger oxidation of cyclohexanones using urea hydrogen peroxide in ethyl acetate (Entry 9, 15, 16, 20). The multimodal pore silica-supported catalyst showed higher protein loads and activity than the SBA-15-based biomaterial. Cyclohexanone was converted to ε-caprolactone in 80% yield, while 2- and 4-methylcyclohexanone gave 97% and 82% yields, respectively, demonstrating the efficiency, stability, and reusability (at least five cycles) of the developed biocatalyst. This report showed that smart support design is crucial to achieve a highly active and stable biocatalytic system [37]. Lipase from *Trichosporon laibacchii* immobilized on diatomite was used for Baeyer–Villiger oxidation of cyclohexanone, giving ε-caprolactone in 98% yield at high concentration (1.2 M) under optimized conditions (cyclohexanone: UHP = 1:1.3, 56.5 °C, ethyl acetate) (Entry 10). These results demonstrate that the use of stable, immobilized lipases can achieve high yields and product concentrations, which are crucial for cost-effective lactone production [38]. A new method was developed using free CALB or Novozyme 435 suspended in ionic liquids (ILs) (Entry 11, 17, 21). Under mild conditions (30% aq. H_2_O_2_, 45 °C), 1-butyl-3-methyl bistriflimide ([bmim][NTf_2_]) was identified as the most effective solvent and enabled an increase in reaction rates compared with toluene. Cyclohexanone was converted to ε-caprolactone in 85% yield, while 2- and 4-methylcyclohexanone gave 99% and 80% yields, respectively, in 10 h. The lipase displayed good stability in the IL, and the IL could be recycled, which demonstrates the potential of ILs to enhance efficiency and sustainability in chemo-enzymatic lactone synthesis [39]. The role of ILs in chemo-enzymatic Baeyer–Villiger oxidation will be described in this review later.

**Scheme 5:**
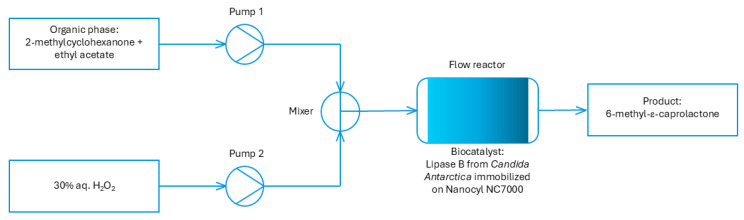
Continuous flow chemo-enzymatic Baeyer–Villiger oxidation of 2-methylcyclohexanone using CALB immobilized on multiwalled carbon nanotubes.

**Scheme 6:**
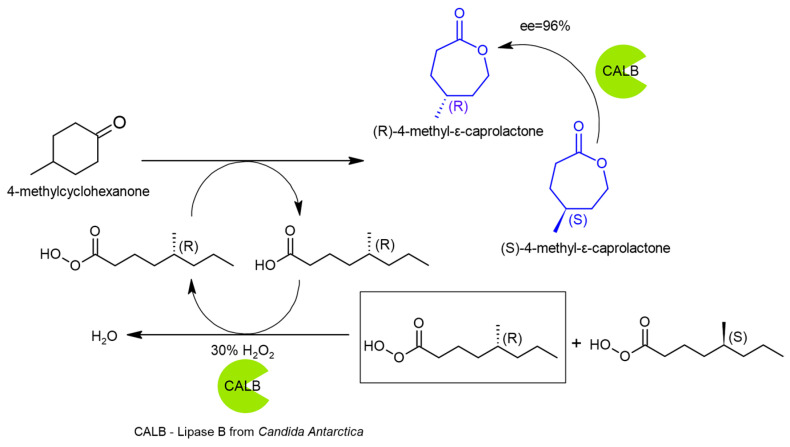
Asymmetric conversion of prochiral 4-methylcyclohexanone to (*R*)-4-methylcaprolactone *via* chemo-enzymatic Baeyer–Villiger oxidation.

An innovative alternative approach was developed for continuous flow chemo-enzymatic Baeyer–Villiger oxidation using CALB immobilized *via* simple physical adsorption on multiwalled carbon nanotubes (Nanocyl NC7000) (Entry 12, 18) (Scheme 5). The nanobiocatalyst generated peracid in situ from ethyl acetate and 30% aq. H_2_O_2,_ providing high yield (90%) and excellent selectivity (>99%) for 2-methylcyclohexanone oxidation to 6-methyl-ε-caprolactone at 40 °C after 60 min [40]. The use of a flow reactor allowed for shorter reaction times, increased safety due to in situ peracid formation, and higher product yields compared with batch processes. This work demonstrates the first application of the developed nanobiocatalyst in continuous flow Baeyer–Villiger oxidation and highlights its potential for efficient, safe, and scalable industrial lactone synthesis. Another research study presented an innovative application of the chemo-enzymatic Baeyer–Villiger oxidation for the asymmetric conversion of prochiral 4-methylcyclohexanone to (*R*)-4-methylcaprolactone using racemic (±)-4-methyloctanoic acid, CALB, and 30% aq. H_2_O_2_ (Entry 22) (Scheme 6). 4-methylcyclohexanone was converted with 97% yield under mild conditions (room temperature, toluene). At the beginning, low enantiomeric excess (ee < 20%) was detected after eight hours, and it then increased to 96% ee after eight days [41].

**Scheme 7:**
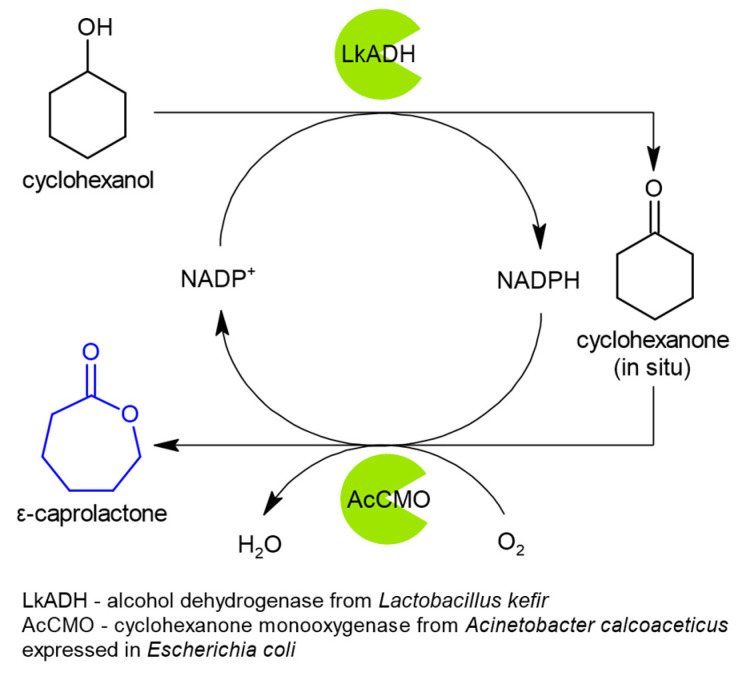
Biosynthesis of ε-caprolactone directly from cyclohexanol.

The biocatalytic synthesis of ε-caprolactone can be achieved from cyclohexanol. It represents a promising approach in green chemistry and relies on microorganisms capable of sequential oxidation. This process typically involves two enzymatic steps in a concurrent cascade: an alcohol dehydrogenase (ADH) catalyzes the oxidation of cyclohexanol to cyclohexanone, which is then converted to ε-caprolactone via Baeyer–Villiger oxidation (Scheme 7). According to the literature, the overall transformation can be considered an oxidative lactonization, in which the initial alcohol is oxidized to an aldehyde or ketone intermediate, which is further oxidized to the lactone. Pioneering studies on ADH-catalyzed oxidative lactonization [46] demonstrated that stereospecific ADH oxidation can yield enantiomerically enriched lactones through kinetic resolution of racemic alcohols or by employing meso substrates. Since then, various research groups have expanded and optimized this approach, highlighting its potential for preparative scale and environmentally friendly production of ε-caprolactone.

**Scheme 8:**
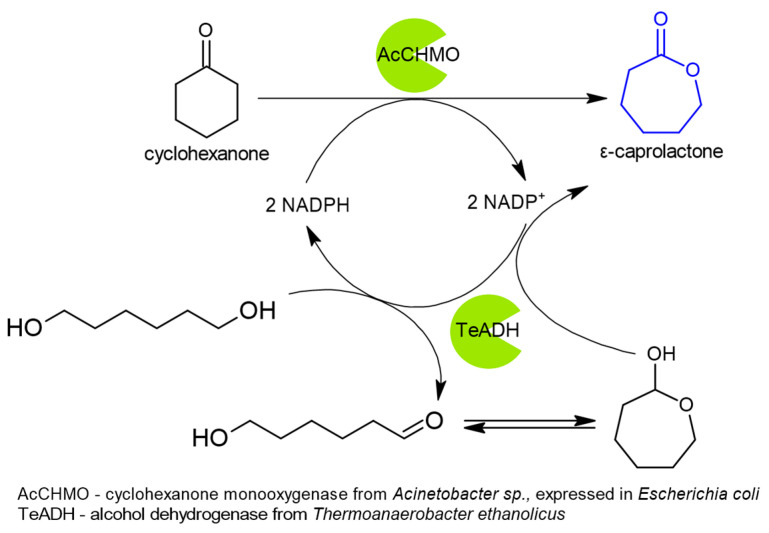
Biosynthesis of ε-caprolactone from 1,6-hexenediol and cyclohexanone.

As an example, ε-caprolactone can be efficiently produced directly from cyclohexanol in a one-pot biocatalytic system using air (Entry 24). In this approach, ADH from *Lactobacillus kefir* works together with a CMO from *Acinetobacter calcoaceticus* expressed in *Escherichia coli*. The system achieved a high conversion (α = 97%) within 24 h using 60 mM cyclohexanol in 5 mL of sodium phosphate buffer (pH 7), with NADP+ added at room temperature. These studies demonstrate that combining these two enzymes in a single pot allows for an efficient and environmentally friendly conversion of cyclohexanol to ε-caprolactone, highlighting the potential of integrated biocatalytic systems for industrial applications [42]. In another study, different reaction conditions were applied (Entry 25). These studies have shown that optimizing reaction conditions can significantly enhance process performance. Performing the reaction with 20 mM cyclohexanol in 200 mM Tris−HCl buffer (pH 8) at 30 °C under a controlled oxygen flow resulted in an 80% yield after 18 h. Compared with the earlier system, which was conducted at lower buffer concentrations, room temperature, and less controlled oxygenation, these conditions improved substrate conversion and enzyme efficiency [43]. Another approach showed that 100 mM cyclohexanol was converted to the lactone via a biocatalytic double oxidation using a recombinant whole-cell catalyst containing an ADH and a CMO mutant (Entry 26). The reaction was carried out in 9 mL of n-butyl acetate at 25 °C, with molecular oxygen as the only oxidant, and gave a yield of 64% after eight hours. Performing the oxidation in a pure organic medium avoided the extraction of the highly water-soluble product and allowed simplified work-up through centrifugal separation of lyophilized cells and solvent removal [44]. These studies demonstrate that ε-caprolactone can be efficiently produced from cyclohexanol using ADH/CMO systems under various conditions. Optimization of buffer, temperature, and oxygen supply improved conversion in aqueous media. Performing the reaction in an organic solvent simplifies product recovery and work-up, highlighting the flexibility and potential of these biocatalytic approaches. It is also possible to synthesize caprolactone from 1,6-hexanediol and cyclohexanone, and to synthesize ε-caprolactone from 1,6-hexanediol and cyclohexanone using a bi-enzymatic concurrent cascade (Entry 27) (Scheme 8). In this approach, an *Escherichia coli* strain expressing a CMO from *Acinetobacter* sp. and an ADH from *Thermoanaerobacter ethanolicus* converts 100 mM cyclohexanone and 50 mM 1,6-hexanediol in 100 mM Tris–HCl buffer (pH 8) with NADP^+^ addition at 30 °C. The system initially achieved a 27% yield in small-scale reactions, while scale-up to 50 mL improved performance significantly, achieving > 99% conversion of cyclohexanone and 20 mM ε-caprolactone within 18 h. This cascade efficiently incorporates cyclohexanone and 1,6-hexanediol into ε-caprolactone, demonstrating the potential of the presented enzymatic strategy for sustainable lactone production [45].

The biosynthesis of ε-caprolactone and its derivatives has been successfully achieved using monooxygenases, lipases, and combinations of alcohol dehydrogenases with Baeyer–Villiger monooxygenases. These studies highlight the critical role of enzyme and microbial engineering, process optimization, and innovative strategies such as smart immobilization and support design in enhancing catalytic efficiency, selectivity, and stability. Careful tuning of reaction conditions, including medium composition, temperature, cofactor regeneration, and oxygen supply, proved to be essential for maximizing production yields. These advances demonstrate how integrated biocatalytic approaches not only improve process performance but also strongly support the principles of green chemistry by minimizing the use of hazardous reagents, reducing waste, and enabling sustainable, environmentally friendly production routes.

## 3. Biosynthesis of Lactones in Ionic Liquids and Deep Eutectic Solvents

Ionic liquids (ILs) are increasingly explored as alternative solvents in biocatalytic reactions due to their exceptional properties, such as negligible vapor pressure, tunable polarity, and high stability under thermal and chemical conditions. ILs offer several advantages over conventional aqueous or organic solvents, such as enhanced solubility of hydrophobic substrates, improved enzyme stability, and the potential for easy product recovery. By providing a tailored microenvironment, ILs can modulate enzyme activity and selectivity, allowing for more efficient and sustainable production of many highly valuable organic compounds. Moreover, the use of ILs aligns with the principles of green chemistry by reducing reliance on volatile organic solvents and enabling the use of recyclable reaction media. ILs can strongly influence enzyme activity and process efficiency in chemo-enzymatic Baeyer–Villiger oxidations by providing a structured reaction environment. Due to their ability to form organized nanoscale domains with distinct polar and nonpolar regions, ILs can preserve the essential hydration layer of enzymes, thereby maintaining their native conformation and catalytic activity. Enzyme-compatible ILs that stabilize the biocatalyst and dissolve poorly soluble substrates are therefore highly attractive for lactone synthesis.

This chapter reviews recent advances in lactone biosynthesis in the presence of ionic liquids, with a focus on the effects of IL structure, enzyme compatibility, and process optimization on reaction efficiency and product yield. Table 2 presents the chemo-enzymatic synthesis of lactones in the presence of ILs.

As an example highlighting the importance of ionic liquids in chemo-enzymatic Baeyer–Villiger oxidation of cyclic ketones, a lipase-catalyzed system using hydrogen-bond-donating ionic liquids and hydrogen peroxide as the oxidant was reported. The ILs played a dual role by enhancing the peracid-mediated oxygen transfer step and by stabilizing CALB through dissolution in the 1-(3-hydroxypropyl)-3-methylimidazolium nitrate and triethanolammonium nitrate. In this system based on Novozyme 435, cyclopentanone, cyclohexanone, and menthone were converted into the corresponding lactones with yields of 99%, 54%, 83%, respectively, at 50 °C after five hours. In comparison, when reactions were conducted in acetonitrile (for cyclopentanone) or ethyl acetate (for cyclohexanone), the yields reached approximately 80%, but at a higher temperature (60 °C) and longer reaction times (24 h or six days, respectively) (Entries 1–5) [47,48]. Other studies presented chemo-enzymatic oxidation of 2-methylcyclohexanone, cyclohexanone, cyclobutanone, and 2-adamantanone to the corresponding lactones in various ionic liquids, mainly based on imidazolium cations and the bis(trifluoromethanesulfonyl)imide [NTf_2_]^−^ anion. The best enzymatic performance and lactone yields were achieved in 1-butyl-3-methylimidazolium bis(trifluoromethanesulfonyl)imide ([bmim][NTf_2_]). Under optimized conditions (cyclohexanone: H_2_O_2_ (1:2, *n*:*n*), octanoic acid, [bmim][NTf_2_], 45 °C), lactone yields of 83–99% were obtained after 10 h of reaction (Entries 6–9). When the reaction was performed in toluene, reduced yields (65–95%) were observed along with prolonged reaction times of up to 20 h [39]. In another approach, the IL was not used as a solvent but as a support modifier to enhance lipase performance. Imidazolium-based ILs were chemically anchored to the multiwalled carbon nanotubes with various anions, such as [Oc_2_PO_4_]^−^, [MeSO_4_]^−^, [I]^−^, [NTf_2_]^−^, and then the activity of three different lipases was tested for the Baeyer–Villiger oxidation of 2-adamantanone (Entries 18–20). Moreover, the IL was incorporated into the support *via* an imine or amide bond. All immobilized lipases exhibited the highest catalytic activity in the presence of the [Oc_2_PO_4_]^−^ anion, giving ketone conversions of 90% (CALB), 93% (lipase from *Aspergillus oryzae*), and 51% (lipase from *Candida rugosa*), with 99% selectivity. Stability tests demonstrated that the presence of an imine bond in the structure stabilizes the ionic liquid and prevents chemical bond hydrolysis under harsh reaction conditions. In addition, when CALB was immobilized on unmodified multiwalled carbon nanotubes, the conversion of 2-adamantanone was significantly lower (44%), which indicates that the presence of ILs on the support is essential for obtaining an active biocatalytic system [49].

**Table 2:** Chemo-enzymatic synthesis of lactones in the presence of ionic liquids.

Entry	Ketone	Enzymatic Catalyst	Ionic Liquid/Deep Eutectic Solvent	Reaction Conditions	Reaction Parameters	Ref.
1	cyclopentanone	Novozyme 435	[HOPMIm][NO_3_] ^a^	0.5 mmol cyclopentanone (0.5 mM), 1 mmol H_2_O_2_, 1 mmol octanoic acid, 1 mL IL, 50 °C, 5 h	Y ^b^ = 99%, S ^c^ = 99%	[47]
2	cyclohexanone	Novozyme 435	[HOPMIm][NO_3_]	0.5 mmol cyclohexanone (0.5 mM), 1 mmol H_2_O_2_, 1 mmol octanoic acid, 1 mL IL, 50 °C, 5 h	Y = 45%	[47]
3	cyclohexanone	Novozyme 435	[TEOA][NO_3_] ^d^	0.5 mmol cyclohexanone (0.5 mM), 1 mmol H_2_O_2_, 1 mmol octanoic acid, 1 mL IL, 50 °C, 5 h	Y = 62%	[47]
4	cyclohexanone	Novozyme 435	[TEOA][NO_3_]	0.5 mmol cyclohexanone (0.5 mM), 1 mmol H_2_O_2_, 1 mmol octanoic acid, 1 mL IL, 50 °C, 5 h	Y = 54%	[47]
5	menthone	Novozyme 435	[HOPMIm][NO_3_]	0.5 mmol menthone (0.5 mM), 1 mmol H_2_O_2_, 1 mmol octanoic acid, 1 mL IL, 50 °C, 5 h	Y = 83%	[47]
6	2-methylcyclohexanone	CALB ^e^	[bmim][NTf_2_] ^f^	2 mmol 2-methylcyclohexanone (0.5 mM), cyclohexanone: H_2_O_2_ (1:2, *n*:*n*), 4 mmol octanoic acid, 4 mL [bmim][NTf_2_], 45 °C, 10 h	Y = 99%	[39]
7	cyclohexanone	CALB	[bmim][NTf_2_]	2 mmol cyclohexanone (0.5 mM), cyclohexanone: H_2_O_2_ (1:2, *n*:*n*), 4 mmol octanoic acid, 4 mL [bmim][NTf_2_], 45 °C, 10 h	Y = 83%	[39]
8	cyclobutanone	CALB	[bmim][NTf_2_]	2 mmol cyclobutanone (0.5 mM), cyclohexanone: H_2_O_2_ (1:2, *n*:*n*), 4 mmol octanoic acid, 4 mL [bmim][NTf_2_], 45 °C, 1.5 h	Y = 99%	[39]
9	2-adamantanone	CALB	[bmim][NTf_2_]	2 mmol 2-adamantanone (0.5 mM), cyclohexanone: H_2_O_2_ (1:2, *n*:*n*), 4 mmol octanoic acid, 4 mL [bmim][NTf_2_], 45 °C, 8 h	Y = 99%	[39]
10	2-(4′-isopropylbenzyl)cyclopentanone	CALB on acrylic resin	Choline chloride:UHP ^g^ (1:2, *n*:*n* ^h^)	25 µmol ketone (0.03 mM), 0.9 mL DES ^i^ 5 mg biocatalyst, 25 µL octanoic acid, 55 °C, 800 rpm	α ^j^ = 99% after 3 days	[50]
11	2-(4′-methylbenzyl)cyclopentanone	CALB immobilized on acrylic resin	Choline chloride:UHP (1:2, *n*:*n*)	25 µmol ketone (0.03 mM), 0.9 mL DES, 5 mg biocatalyst, 25 µL octanoic acid, 55 °C, 800 rpm	α = 99% after 3 days	[50]
12	2-benzylcyclopentanone	CALB immobilized on acrylic resin	Choline chloride:UHP (1:2, *n*:*n*)	25 µmol ketone (0.03 mM), 0.9 mL DES, 5 mg biocatalyst, 25 µL octanoic acid, 55 °C, 800 rpm	α = 92% after 3 days	[50]
13	2-(4′-isopropylbenzyl)cyclopentanone	CALB immobilized on acrylic resin	Choline chloride:Urea (1:2, *n*:*n*) and H_2_O_2_ (10 wt%)	25 µmol ketone (0.03 mM), 0.9 mL DES, 5 mg biocatalyst, 25 µL octanoic acid, 55 °C, 800 rpm	α = 98% after 3 days	[50]
14	2-(4′-′methylbenzyl)cyclopentanone	CALB immobilized on acrylic resin	Choline chloride:Urea (1:2, *n*:*n*) and H_2_O_2_ (10 wt%)	25 µmol ketone (0.03 mM), 0.9 mL DES, 5 mg biocatalyst, 25 µL octanoic acid, 55 °C, 800 rpm	α = 95% after 3 days	[50]
15	2-benzylcyclopentanone	CALB immobilized on acrylic resin	Choline chloride:Urea (1:2, *n*:*n*) and H_2_O_2_ (10 wt%)	25 µmol ketone (0.03 mM), 0.9 mL DES, 5 mg biocatalyst, 25 µL octanoic acid, 55 °C, 800 rpm	α = 91% after 3 days	[50]
16	2-benzylcyclopentanone	CALB immobilized on acrylic resin	Choline chloride:Glucose (2:1, *n*:*n*) and H_2_O_2_ (30 wt%)	25 µmol ketone (0.03 mM), 0.9 mL DES, 5 mg biocatalyst, 25 µL octanoic acid, 55 °C, 800 rpm	α = 70–90% after 3–6 days	[50]
17	2-benzylcyclopentanone	CALB immobilized on acrylic resin	Choline chloride:Glycerol (1:2, *n*:*n*) and H_2_O_2_ (10 wt%)	25 µmol ketone (0.03 mM), 0.9 mL DES, 5 mg biocatalyst, 25 µL octanoic acid, 55 °C, 800 rpm	No conversion	[50]
18	2-adamantanone	CALB immobilized on SILLP	Imidazolium-based IL with [Oc_2_PO_4_]^−^) ^k^ anion	1 mmol 2-adamantanone (1 mM), 0.5 mL toluene, 0.5 mL n-octanoic acid, 2 mmol 35% aq. H_2_O_2_, 20 °C, 4 h, 400 rpm	α = 90%, S = 99%	[49]
19	2-adamantanone	Lipase from *Aspergillus oryzae* immobilized on SILLP	Imidazolium-based IL with [Oc_2_PO_4_]^−^) anion	1 mmol 2-adamantanone (1 mM), 0.5 mL toluene, 0.5 mL n-octanoic acid, 2 mmol 35% aq. H_2_O_2_, 20 °C, 4 h, 400 rpm	α = 93%, S = 99%	[49]
20	2-adamantanone	Lipase from *Candida rugosa* immobilized on SILLP	Imidazolium-based IL with [Oc_2_PO_4_]^−^) anion	1 mmol 2-adamantanone (1 mM), 0.5 mL toluene, 0.5 mL n-octanoic acid, 2 mmol 35% aq. H_2_O_2_, 20 °C, 4 h, 400 rpm	α = 51%, S = 99%	[49]
21	cyclohexanone	CALB	Choline chloride:Sorbitol (1:1, *n*:*n*)	1 mmol cyclohexanone (1 mM), 2 mmol 30% aq. H_2_O_2_, 1 mmol octanoic acid, DES 1.2 g, 0.3 mL H_2_O, 40 °C, 48 h	α = 92%, S = 46%	[51]
22	cyclopentanone	CALB	Choline chloride:Sorbitol (1:1, *n*:*n*)	1 mmol cyclopentanone (1 mM), 2 mmol 30% aq. H_2_O_2_, 1 mmol octanoic acid, DES 1.2 g, 0.3 mL H_2_O, 40 °C, 48 h	α = 95%, S = 48%	[51]
23	cyclohexanone	Ser105Ala	Choline chloride:Sorbitol (1:1, *n*:*n*)	1 mmol cyclohexanone (1 mM), 2 mmol 30% aq. H_2_O_2_, 1 mmol octanoic acid, DES 1.2 g, 0.3 mL H_2_O, 40 °C, 48 h	α = 47%, S = 97%	[51]
24	cyclopentanone	Ser105Ala	Choline chloride:Sorbitol (1:1, *n*:*n*)	1 mmol cyclopentanone (1 mM), 2 mmol 30% aq. H_2_O_2_, 1 mmol octanoic acid, DES 1.2 g, 0.3 mL H_2_O, 40 °C, 48 h	α = 51%, S = 99%	[51]

[^a^] 1-(3-hydroxypropyl)-3-methylimidazolium nitrate. [^b^] yield. [^c^] selectivity. [^d^] triethanolammonium nitrate. [^e^] Lipase B from *Candida antarctica*. [^f^] 1-butyl-3-methylimidazolium bis(trifluoromethanesulfonyl)imide. [^g^] urea hydrogen peroxide. [^h^] molar ratio. [^i^] deep eutectic solvent. [^j^] conversion. [^k^] dioctyl phosphate anion.

Reports describing the use of ILs in chemo-enzymatic Baeyer–Villiger oxidation are still limited. Therefore, selected examples employing deep eutectic solvents (DESs) are also discussed, as these media share many features with ILs and represent green and sustainable alternatives to conventional solvents. However, reports on their application in Baeyer–Villiger oxidation remain limited as well. For example, chemo-enzymatic Baeyer–Villiger oxidation of α-benzylcyclopentanones was investigated in ester solvents and DESs. For DESs in which the oxidant was not incorporated into the solvent structure, no oxidation reaction was observed. In contrast, positive effects were achieved for DESs containing the oxidant within their structure, mainly those based on urea and sugars. No conversion was detected for glycine-based DESs. The reaction showed the highest efficiency in minimal DESs that included the oxidant, enabling conversions exceeding 92% within three days. The results obtained for ethyl acetate as a reaction solvent were comparable to those observed in DESs (Entries 10–17). A key advantage of DESs is that the solvent itself functions as the oxidant, thereby simplifying the overall reaction system. Notably, choline chloride: UHP (1:2, *n*:*n*) DES provided the highest reaction rate for ketone conversion to the corresponding lactone, leading to a green, efficient process [50]. Another report showed the great potential of Ser105Ala, a mutant of the CALB, enabling „perhydrolase-only” reactions (Entry 21–24). In comparison to CALB, Ser105Ala enabled selective oxidation of, e.g., cyclohexanone in choline chloride: sorbitol (1:1, *n*:*n*) by eliminating the undesired hydrolysis reaction of the product (97% selectivity, 47% conversion). For tests conducted in the water: n-hexane (1:2, *v*:*v*) system in the presence of CALB or the Ser105 variant, a decrease in both conversion and selectivity was observed [51].

**Figure 2:**
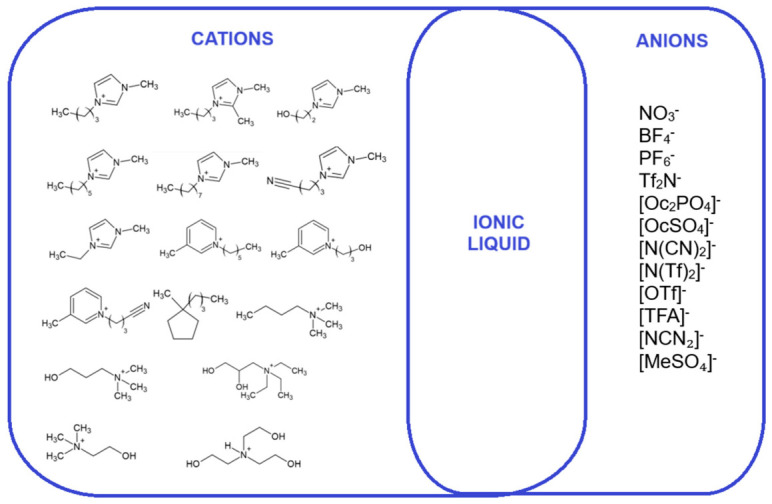
Cations and anions of ionic liquids used in chemo-enzymatic Baeyer–Villiger oxidation.

ILs play a crucial role in the biocatalytic synthesis of lactones by modulating enzyme stability, activity, and overall process efficiency. The nature of the anion has been identified as a key factor determining lipase activity: hydrophobic anions such as [NTf_2_]^−^ provide the highest activity in Baeyer–Villiger oxidations of cyclic ketones, likely by preserving flexibility in the active conformation of the enzyme, whereas other hydrophobic anions, e.g., [PF_6_]^−^, exhibit low or no activity, probably due to enzyme deactivation caused by hydrolysis-derived toxic species. Hydrophilic anions negatively affect the reaction, likely by attracting essential water from the enzyme and promoting protein unfolding, which confirms the dominant role of the anion in controlling enzyme stability and catalytic efficiency. The structure of the cation also strongly influences the performance of enzymes. Among different cations, dialkylimidazolium-based ILs showed the best activity, with 1-butyl-3-methylimidazolium ([bmim]^+^) giving optimal results for native CALB. Substitution at the C2 position of the imidazolium ring reduced activity, indicating that hydrogen bond interactions between the cation and the enzyme can interfere with efficient catalysis. Comparable activity was observed for pyridinium- and ammonium-based cations, while pyrrolidinium cations resulted in lower reaction rates. Functionalization of the cation with –OH or –CN groups further modulated enzyme activity: hydroxylated cations generally decreased activity for native CALB due to increased viscosity in highly functionalized ILs, which limited the kinetics of the studied reaction. Figure 2 presents the cations and anions that were tested in chemo-enzymatic Baeyer–Villiger oxidations.

Beyond their use as solvents, ILs can also serve as support modifiers to enhance enzyme performance. Imidazolium-based ILs chemically anchored to multiwalled carbon nanotubes via imine or amide bonds and bearing different anions ([Oc_2_PO_4_]^−^, [MeSO_4_]^−^, [I]^−^, [NTf_2_]^−^) were shown to significantly improve the activity of immobilized lipases in the Baeyer–Villiger oxidation of 2-adamantanone. Among these, the [Oc_2_PO_4_]^−^ anion provided the highest catalytic activity, achieving 90% ketone conversion for CALB, 93% for lipase from *Aspergillus oryzae*, and 51% for lipase from *Candida rugosa* (with 99% selectivity). Stability tests revealed that the presence of an imine bond in the IL-support structure stabilized the IL and prevented hydrolysis under harsh reaction conditions, thereby enhancing biocatalyst performance.

Overall, these studies demonstrate that both anion and cation structures, as well as the method of IL incorporation (as a solvent or support modifier), critically determine enzyme compatibility, activity, and productivity in Baeyer–Villiger oxidations conducted in ILs. Rational selection and design of ILs allow designing the microenvironment of the enzyme, including projectable solubility, viscosity, hydrogen bond interactions, and stabilization of essential water. Such strategies provide powerful tools for optimizing chemo-enzymatic lactone synthesis, enabling efficient, selective, and sustainable biocatalytic processes that align with the principles of green chemistry.

## 4. Green Chemistry Metrics Evaluation

To evaluate the sustainability profile of biocatalytic Baeyer–Villiger oxidation of cyclic ketones performed in non-conventional media, the examples summarized in Table 2 were further analyzed using J. Clark’s Green Chemistry Metrics toolkit (File S1, Supplementary Materials) [52]. A total of twenty-four reactions were selected for comparative assessment (Table 3). All analyzed systems relied on lipase-catalyzed in situ generation of peroctanoic acid from octanoic acid in the presence of hydrogen peroxide or UHP as the oxidant. The dataset considered in this section was intentionally limited to reactions conducted in alternative media, namely, ILs and DESs, in order to assess the sustainability potential of these systems within the context of green chemistry principles.

The analyzed systems presented a diverse range of biocatalysts: Novozyme 435 (entries 1′–5′), free CALB (entries 6′–9′), CALB immobilized on acrylic resin (entries 10′–17′), lipases supported on SILLP materials (entries 18′–20′), as well as the catalytically modified Ser105Ala CALB mutant (entries 21′–24′). The substrate scope included cyclobutanone, cyclopentanone, cyclohexanone, 2-methylcyclohexanone, menthone, 2-adamantanone, and a series of substituted 2-arylmethylcyclopentanones, providing a relatively broad basis for comparing catalytic efficiency and sustainability indicators across structurally distinct substrates.

In most cases, lactone selectivity remained high (97–100%), indicating that lipase-mediated peracid in situ generation enables remarkably clean Baeyer–Villiger oxidation with only limited side-product formation. Such selectivity is particularly significant from a green chemistry perspective, as it minimizes downstream purification requirements and reduces waste generation associated with undesired by-products. Nevertheless, two notable exceptions are worth highlighting. Native CALB operating in ChCl DES (entries 21′–22′) provided high conversion values (92–95%), but poor selectivity (46–48%). The introduction of the Ser105Ala mutant significantly improved selectivity (97–99%, entries 23′–24′); however, this improvement was accompanied by a noticeable decrease in conversion to 47–51%. The improved selectivity observed for the Ser105Ala variant was associated with lower conversion, a phenomenon frequently reported in enzyme engineering, where mutations that suppress competing reaction pathways may also reduce overall catalytic efficiency. In contrast, no conversion was detected in the ChCl DES system (entry 17′), despite similar reaction conditions to those in entries 15′ and 16′. This observation highlights the importance of DES composition and shows that the performance of the biocatalyst strongly depends on the choice of hydrogen-bond donor.

Atom economy (AE) values were determined by substrate structure and ranged from 40.2% for cyclobutanone (entry 8′) to 64.4% for 2-(4′-isopropylbenzyl)cyclopentanone (entries 10′ and 13′). The moderate AE values observed throughout the dataset arise from the intrinsic stoichiometry of Baeyer–Villiger oxidation, in which one equivalent of water is inevitably generated during oxygen insertion. Consequently, even an ideal catalytic system cannot exceed approximately 65% AE for this transformation.

Reaction mass efficiency (RME), which is directly dependent on isolated yield and the stoichiometric excess of reagents, varied across the dataset from 7.6% to 43.3%. The highest RME values were obtained for native CALB in ChCl:Sorbitol DES (entries 21′–22′, 41.7–43.3%), but as discussed above, these arise from poor selectivity rather than from an efficient use of mass and should therefore not be interpreted as a sustainability advantage. For SILLP-supported lipases (entries 18′–20′), RME reached 14.0–25.5%, with the highest value recorded for lipase from Aspergillus oryzae immobilized on SILLP (entry 19′, 25.5%). This confirmed the favorable role of the hydrophobic IL microenvironment in maintaining lipase activity. The lowest RME values (7.6–11.2%) were observed for CALB immobilized on acrylic resin in choline chloride-based DES during arylmethylcyclopentanone oxidations (entries 10′–17′). This is mainly due to the high mass contribution of the DES components. However, this does not indicate lower sustainability, particularly when the DES can be recovered and reused.

All twenty-four entries use non-conventional reaction media. The dataset can be divided into three subclasses, which were evaluated separately using the toolkit. The first group comprised protic ionic liquids such as [HOPMIm][NO_3_] and [TEOA][NO_3_] (entries 1′–5′). The presence of hydrogen-bonding functionalities in the cation allows these ILs to form extended hydrogen-bond networks, which may partially mimic the structuring role of water in enzyme environments [23,53]. Their negligible vapor pressure addresses one of the main limitations of volatile organic solvents. However, limited information on biodegradability and environmental impact prevents their clear classification as green solvents, resulting in an amber assessment in the toolkit. A second category involved hydrophobic aprotic ILs represented by [bmim][NTf_2_] (entries 6′–9′). This solvent is well known for its compatibility with lipase catalysis and provided high isolated yields across all analyzed cases (83–99%). Its hydrophobic character helps maintain the enzyme hydration layer and stabilize the active conformation of the protein [23,53]. However, the fluorinated [NTf_2_]^−^ anion raises environmental concerns due to poor biodegradability and likely long-term persistence in the environment. The third group comprised SILLPs materials (entries 18′–20′), where an imidazolium-based IL containing the [Oc_2_PO_4_]^−^ anion was grafted to the solid support and used as a matrix for enzyme immobilization, while toluene served as the reaction medium. In this system, the IL layer provides a stabilizing microenvironment for the enzyme and helps to maintain its activity during the reaction. The heterogeneous form of the lipase also enables effortless separation and potential reuse of the catalytic system after the reaction. However, the use of toluene as a cosolvent limits the overall greenness of the process. The largest subgroup involved DES-based systems (entries 10′–17′ and 21′–24′). Five DES formulations were reported, including ChCl:UHP (1:2, entries 10′–12′), in which the urea–hydrogen peroxide adduct serves simultaneously as hydrogen-bond donor and oxidant; ChCl:Urea (1:2) combined with aqueous H_2_O_2_ (entries 13′–15′); ChCl:Glucose (2:1) with H_2_O_2_ (entry 16′); ChCl:Glycerol (1:2) with H_2_O_2_ (entry 17′); and ChCl:Sorbitol (1:1) with H_2_O_2_ (entries 21′–24′). DES systems based on choline chloride and naturally derived hydrogen-bond donors such as urea, glucose, glycerol, or sorbitol fulfill the key criteria of greener reaction media, namely, low toxicity, low volatility, biodegradability, simple preparation and use of inexpensive bio-derived components. Of interest is the ChCl:UHP system (entries 10′–12′), where the DES simultaneously functions as both the reaction medium and the oxidant reservoir. Such dual functionality represents an example of process intensification through solvent design and demonstrates how reaction media can actively contribute to overall process efficiency instead of only providing the reaction medium.

A green flag for catalyst use was assigned to all twenty-four entries, since the lipase is a renewable, biodegradable, and non-toxic catalyst that contains no critical elements. Regarding catalyst recovery, immobilized systems (entries 6′–20′; Novozyme 435, CALB on acrylic resin, and SILLP-based biocatalysts) were flagged green, as the heterogeneous biocatalyst can be filtered off and reused in subsequent cycles. Native lipases used in DES systems (entries 21′–24′) received an amber flag, mainly due to limited catalyst recovery options. This is partly mitigated by the possibility of recycling the DES phase, although no recyclability data were reported in the analyzed studies. As a result, a full assessment of the long-term environmental impact is not possible.

In the studied examples, reaction temperatures ranged from 20 to 55 °C, remaining well below the decomposition temperatures of all employed media and supporting favorable scores for energy demand and thermal safety. Some DES-based systems required longer reaction times, up to several days, to achieve satisfactory conversion. Although this does not necessarily imply high energy consumption, it may limit process productivity and industrial attractiveness. Notably, every example identified in the literature relied on batch processing. To date, no continuous flow biocatalytic Baeyer–Villiger oxidation in either ILs or DES has been reported. Given the well-recognized advantages of flow processing for oxidation chemistry, including improved heat and mass transfer as well as safer oxidant handling, this area appears particularly promising for future development.

The Health and Safety assessment was dominated by the use of hydrogen peroxide, which was employed as the stoichiometric oxidant in all analyzed reactions. Hydrogen peroxide, due to its hazardous properties, was the main factor responsible for the red flag assigned across the dataset, regardless of the reaction medium used. In comparison, the solvents reported for extraction and analysis had a smaller impact on the overall assessment. Ethyl acetate was used in the DES-based studies, whereas dichloromethane or hexane were reported in several IL-based protocols.

Overall, the green metrics analysis indicates that biocatalytic Baeyer–Villiger oxidation performed in ILs and DES systems represents a promising alternative to conventional oxidation methods. Most systems provided high selectivity and satisfactory conversions under mild reaction conditions, benefiting from the use of renewable biocatalysts and non-volatile reaction media. Among the evaluated solvents, DES-based systems showed the most favorable sustainability profile due to the use of readily available and biodegradable components, while the environmental impact of several ILs was limited by concerns related to the imidazolium cation or fluorinated anions. The analysis also highlights several limitations. Insufficient information on solvent and catalyst recycling prevented a comprehensive assessment of long-term process sustainability. Across the entire dataset, hydrogen peroxide was identified as the principal factor affecting the Health and Safety score, regardless of the reaction medium used.

## 5. Conclusions

Ionic liquids play an important role in the biocatalytic synthesis of lactones. They strongly influence enzyme stability, activity, and process efficiency. Both solvent-based and support-modifying applications show that the anion is a key factor in lipase performance. Hydrophobic anions, such as [NTf_2_]^−^, preserve enzyme flexibility and activity. Hydrophilic or hydrolytically unstable anions can deactivate the enzyme. Using IL-modified supports enhances catalytic activity and stability for different lipases, especially when the IL is anchored through stable bonds. The cation structure also affects enzyme behavior through hydrogen bonding and viscosity. These results show that careful design of both the anion and the cation is essential. Overall, ionic liquids act as tunable functional materials rather than inert solvents. They are powerful tools for optimizing Baeyer–Villiger oxidations and supporting sustainable lactone production. Among the different families of ILs, imidazolium- and chloride-based ILs remain the most widely used in lipase-catalyzed lactone synthesis due to their tunable structure and relatively well-characterized physicochemical properties. However, their application is limited, as certain combinations of imidazolium alkyl chain length and anion type may lead to reduced activity of the enzyme or its partial deactivation. More hydrophobic ILs can preserve enzyme flexibility, whereas highly hydrophilic or reactive anions may negatively affect the stable conformation of the lipase. Alternatively, more recently developed amino acid-based ILs are gaining attention as potentially more biocompatible and environmentally friendly media, but their application in Baeyer–Villiger oxidations remains largely explored [54]. Looking forward, further advances in this field are expected to arise from the discovery and development of novel lipases with improved stability and selectivity in ionic liquids. Additionally, protein engineering strategies to tailor enzyme active sites would enhance performance in specific IL environments. Designing ILs to favor enantioselective transformations could enable the efficient synthesis of enantiomerically pure lactones and their derivatives, which is important for the pharmaceutical sector. Integration of these approaches with scalable immobilization techniques and process intensification is likely to expand the applicability of IL-based systems, paving the way for greener, more sustainable, and industrially relevant production of high-value lactones. In the future, continuous flow synthesis and the development of novel IL-based supports are expected to play a key role in further improving the efficiency, selectivity, and scalability of lactone production.

## Figures and Tables

**Table 1:** Biosynthesis of ε-caprolactone and its structurally related alkyl derivatives.

Entry	Starting Material	Enzymatic Catalyst	Oxidizing Agent	Reaction Conditions	Reaction Parameters	Ref.
1	Cyclohexanone	*Escherichia coli* strain expressing *Acinetobacter* sp. CMO ^a^	O_2_	90 mmol cyclohexanone, 6 Lmin^−1^ air flow, M9 Minimal Salts, 30 mM glucose, pH = 7, 32 °C	Y ^b^ = 88% (13 h)	[29]
2		*Escherichia coli* expressing *Acinetobacter calcoaceticus* CMO	O_2_	61 mM cyclohexanone, 3.5 gL^−1^ sodium–potassium phosphate buffer, pH = 7, 5 gL^−1^ glucose, 30 °C	Y = 65% (38 h)	[30]
3		*Corynebacterium glutamicum* expressing *chnB* of *Acinetobacter calcoaceticus* CMO	O_2_	55 mM cyclohexanone, Riesenberg medium including 25 g mL^−1^ kanamycin, 25% ammonium hydroxide, 10% dissolved oxygen tension, pH = 6.8, 30 °C	Y = 94% (7 h)	[31]
4		*Geotrichum candidum* NCYC49 strain immobilized on polyacrylamide	O_2_	5 mM cyclohexanone, 50 mM KH_2_PO_4_ buffer, pH = 6.5, 28 °C	Y = 99% (72 h)	[32]
5		*Geotrichum candidum* whole cells CCT1205 (1 g)	O_2_	10 µL cyclohexanone (9.65 mM), 10 mL phosphate buffer, pH = 6.5, 28 °C, 180 rpm	Y = 100% (4 h), Recycled 6 times	[33]
6		*Chlamydomonas reinhardtii* whole cells	O_2_	5 mM cyclohexanone, 1.7% (*v*:*v*) ethanol, 26 μmol photons m^−2^s^−1^	Y = 81%, S ^c^ = 81% (19% to cyclohexanol)	[34]
7		Novozyme 435	UHP ^d^	0.5 mmol cyclohexanone (0.33 mM), cyclohexanone: UHP (1:1.1, *n*:*n* ^e^), 1.5 mL ethyl acetate, rt	Y = 80% (6 days)	[35]
8		CALB ^f^ immobilized as CLEAs ^g^	50% aq. H_2_O_2_	0.5 mmol cyclohexanone (0.5 mM), cyclohexanone: H_2_O_2_ (1:2, *n*:*n*), 1 mL ethyl acetate, 40 °C	Y = 79% (48 h)	[36]
9		CALB immobilized on silica material (20 mg)	UHP	1 mmol cyclohexanone (0.33 mM), cyclohexanone: UHP (1:2, *n*:*n*), 3 mL ethyl acetate, rt	Y = 80% (75 h)	[37]
10		Lipase from *Trichosporon laibacchii* immobilized on diatomite support (25 mgmL^−1^)	UHP	1.22 M cyclohexanone, cyclohexanone: UHP (1:1.3, *n*:*n*), ethyl acetate, 56.5 °C, rt	Y = 98.06%	[38]
11		CALB (100 mg)	30% aq. H_2_O_2_	2 mmol cyclohexanone (0.5 mM), cyclohexanone: H_2_O_2_ (1:2, *n*:*n*), 4 mmol octanoic acid, 4 mL [bmim][NTf_2_] ^h^, 45 °C	Y = 85% (15 h)	[39]
12		CALB immobilized on Nanocyl NC7000	30% aq. H_2_O_2_	0.5 mM 2-methyl-cyclohexanone, 0.91 mM H_2_O_2_, cyclohexanone: H_2_O_2_ (1:3, *n*:*n*), ethyl acetate, 0.076 mL/min flow rate, 40 °C	α ^i^ = 83% (60 min)	[40]
13	2-Methyl-cyclohexanone	Novozyme 435	UHP	0.5 mmol 2-methyl-cyclohexanone, cyclohexanone: UHP (1:2, *n*:*n*), 1.5 mL ethyl acetate, rt	Y = 95% (3 days)	[35]
14		CALB immobilized as CLEAs	50% aq. H_2_O_2_	0.5 mmol 2-methyl-cyclohexanone (0.33 mM), cyclohexanone: H_2_O_2_ (1:2, *n*:*n*), 1 mL ethyl acetate, 40 °C	Y = 84% (48 h)	[36]
15		CALB immobilized on silica material (20 mg)	60% aq. H_2_O_2_	1 mmol 2-methyl-cyclohexanone (0.33 mM), cyclohexanone: UHP (1:2, *n*:*n*), 3 mL ethyl acetate, rt	Y = 97% (21 h)	[37]
16		CALB immobilized on silica material (20 mg)	UHP	1 mmol 2-methyl-cyclohexanone (0.33 mM), cyclohexanone: UHP (1:2, *n*:*n*), 3 mL ethyl acetate, rt	Y = 97% (29 h) Recycled 5 times	[37]
17		CALB (100 mg)	30% aq. H_2_O_2_	2 mmol 2-methyl-cyclohexanone (0.5 mM), cyclohexanone: H_2_O_2_ (1:2, *n*:*n*), 4 mmol octanoic acid, 4 mL [bmim][NTf_2_], 45 °C	Y = 99% (10 h)	[39]
18		CALB immobilized on Nanocyl NC7000 (500 mg)	30% aq. H_2_O_2_	0.5 mM 2-methyl-cyclohexanone, 0.91 mM H_2_O_2_, cyclohexanone: H_2_O_2_ (1:3, *n*:*n*), ethyl acetate, 0.076 mL/min flow rate, 40 °C	α = 90% (60 min)	[40]
19	4-Methyl-caprolactone	CALB immobilized as CLEAs	50% aq. H_2_O_2_	0.5 mmol 4-methyl-cyclohexanone (0.5 mM), cyclohexanone: H_2_O_2_ (1:2, *n*:*n*), 1 mL ethyl acetate, 40 °C	Y = 77% (48 h)	[36]
20		CALB immobilized on silica material (20 mg)	UHP	1 mmol 4-methyl-cyclohexanone (0.33 mM), cyclohexanone: UHP (1:2, *n*:*n*), 3 mL ethyl acetate, rt	Y = 82% (72 h)	[37]
21		CALB (100 mg)	30% aq. H_2_O_2_	2 mmol 2-methyl-cyclohexanone (0.5 mM), cyclohexanone: H_2_O_2_ (1:2, *n*:*n*), 4 mmol octanoic acid, 4 mL [bmim][NTf_2_], 45 °C	Y = 80% (10 h)	[39]
22		CALB (100 mg)	30% aq. H_2_O_2_	0.5 mmol cyclohexanone (0.5 mM), cyclohexanone: H_2_O_2_ (1:2, *n*:*n*), 1 mmol (±)-4-methyloctanoic acid, 1 mL toluene, rt	Y = 97% (8 h, ee < 20%; 8 days, ee = 96%)	[41]
23	Cyclohexanol	*Geotrichum candidum* whole cells CCT1205 (1 g)	O_2_	10 µL cyclohexanol (9.65 mM), 10 mL phosphate buffer, pH = 6.5, 28 °C, 180 rpm	Y = 100% (4 h)	[33]
24		ADH ^j^ from *Lactobacillus kefir* and *Escherichia coli* expressing *Acinetobacter calcoaceticus* CMO	O_2_	60 mM cyclohexanol, 5 mL sodium phosphate buffer (pH = 7), 1 mg MgCl_2_, 0.002 mmol NADP+, rt	α = 97% (24 h)	[42]
25		ADH from *Lactobacillus kefir* and *Escherichia coli* expressing *Acinetobacter calcoaceticus* CMO	O_2_	20 mM cyclohexanol, 200 mM Tris−HCl, pH = 8, 30 °C, 2 vvm oxygen flow	Y = 80% (18 h)	[43]
26		ADH from *Lactobacillus kefir* and *Escherichia coli* expressing *Acinetobacter* sp. CMO (lyophilized cells)	O_2_	100 mM cyclohexanol, 9 mL n-butyl acetate, 25 °C	Y = 64% (8 h)	[44]
27	1,6-Hexenediol and Cyclohexanone	*Escherichia coli* strain expressing *Acinetobacter* sp. CMO and ADH from *Thermoanaerobacter ethanolicus*	O_2_	100 mM cyclohexanone, 50 mM 1,6-hexenediol, 100 mM Tris−HCl, pH = 8, 1 mM NADP+, 30 °C, 300 rpm	Y = 27%	[45]
28	Cyclohexenone	*Geotrichum candidum* whole cells CCT1205 (3 g)	O_2_	10 µL cyclohexenone (9.65 mM), 10 mL phosphate buffer, pH = 6.5, 28 °C, 180 rpm	Y = 100% (6 h)	[33]

[^a^] cyclohexanone monooxygenase. [^b^] yield. [^c^] selectivity. [^d^] urea hydrogen peroxide. [^e^] molar ratio. [^f^] lipase B from *Candida antarctica*. [^g^] cross-linked enzyme aggregates. [^h^] 1-butyl-3-methylimidazolium bis(trifluoromethanesulfonyl)imide. [^i^] conversion. [^j^] alcohol dehydrogenase.

**Table 3:** Green Chemistry Metrics evaluated for biocatalytic Baeyer–Villiger oxidation of cyclic ketones performed in non-conventional media.

Catalyst/ Reagent	Entry	Ketone	Yield of Product [%]	Selectivity of the BV Oxidation [%]	AE ^a^ [%]	RME ^b^ [%]	Solvent Choice	Catalyst or Reagent	Recoverable Catalyst	Critical Elements	Energy	Reaction Run Below Solvent Boiling Point	Batch/ Flow	Work-Up	Health and Safety	Ref.
Novozyme 435	1′	Cyclopentanone	99	99	43.8	26.6	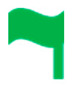	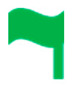	No data	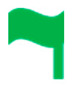	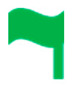	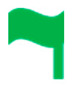	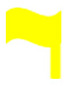	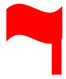	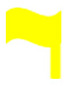	[47]
Novozyme 435	2′	Cyclohexanone	45	99	47.1	13.3	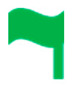	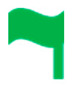	No data	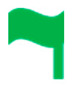	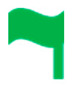	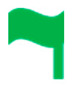	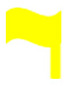	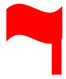	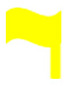	[47]
Novozyme 435	3′	Cyclopentanone	61.9	98.7	43.8	16.6	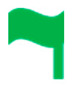	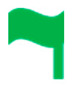	No data	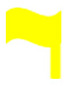	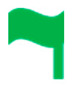	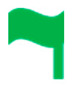	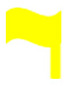	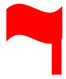	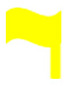	[47]
Novozyme 435	4′	Cyclohexanone	53.9	98.8	47.1	15.9	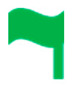	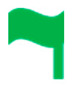	No data	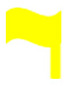	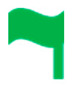	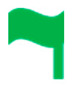	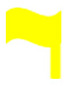	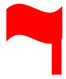	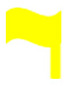	[47]
Novozyme 435	5′	Menthone	82	100	57	31.6	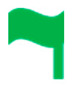	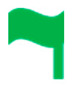	No data	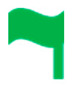	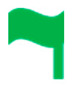	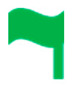	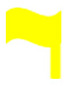	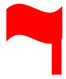	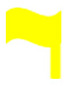	[47]
CALB ^c^	6′	2-Methylcyclohexanone	99	100	50	31.7	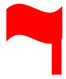	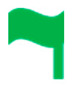	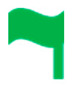	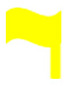	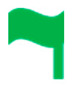	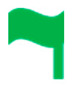	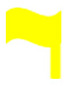	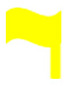	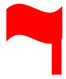	[39]
CALB	7′	Cyclohexanone	83	100	47.1	24.5	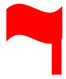	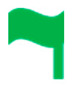	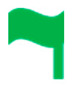	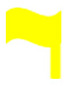	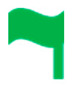	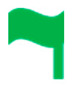	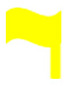	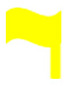	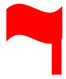	[39]
CALB	8′	Cyclobutanone	99	100	40.2	23.8	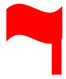	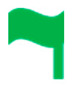	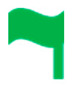	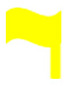	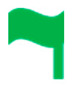	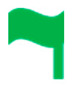	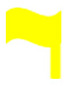	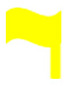	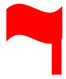	[39]
CALB	9′	2-Adamantanone	99	100	56.5	37.5	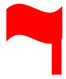	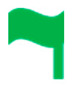	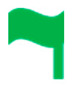	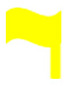	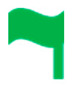	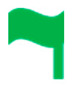	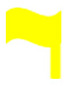	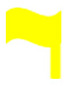	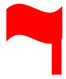	[39]
CALB immobilized on acrylic resin	10′	2-(4′-Isopropylbenzyl)cyclopentanone	99	100	64.4	11.2	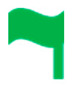	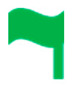	No data	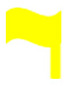	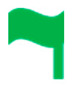	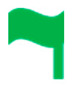	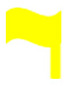	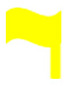	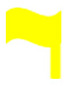	[48]
CALB immobilized on acrylic resin	11′	2-(4′-Methylbenzyl)cyclopentanone	100	100	61.8	10	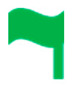	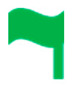	No data	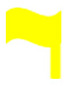	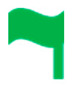	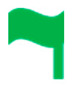	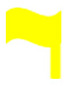	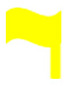	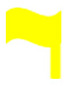	[48]
CALB immobilized on acrylic resin	12′	2-benzylcyclopentanone	92	100	59.7	8.7	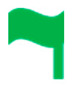	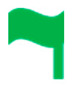	No data	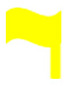	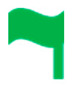	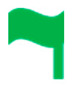	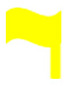	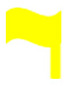	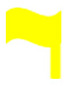	[48]
CALB immobilized on acrylic resin	13′	2-(4′-Isopropylbenzyl)cyclopentanone	98.1	100	64.4	11.1	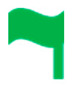	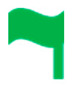	No data	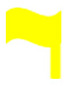	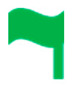	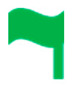	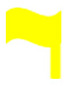	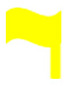	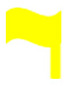	[48]
CALB immobilized on acrylic resin	14′	2-(4′-Methylbenzyl)cyclopentanone	96.2	100	61.8	9.6	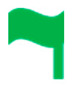	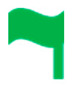	No data	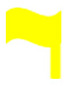	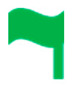	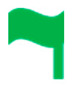	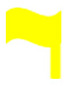	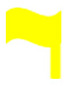	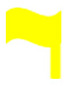	[48]
CALB immobilized on acrylic resin	15′	2-Benzyl- cyclopentanone	91	100	59.7	8.7	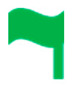	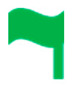	No data	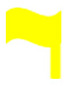	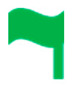	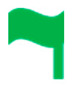	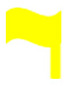	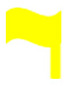	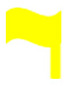	[48]
CALB immobilized on acrylic resin	16′	2-Benzyl- cyclopentanone	79.9	100	59.7	7.6	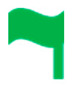	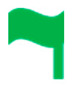	No data	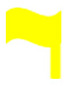	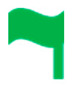	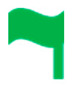	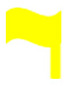	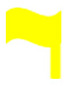	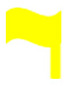	[48]
CALB immobilized on acrylic resin	17′	2-Benzyl- cyclopentanone	-	-	-	-	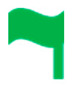	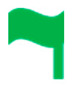	No data	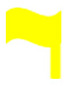	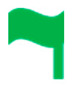	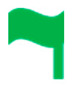	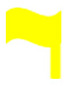	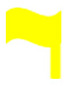	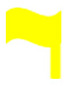	[48]
CALB immobilized on SILLP ^d^	18′	2-Adamantanone	90	99	56.5	24.7	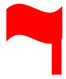	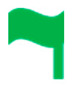	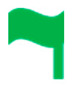	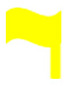	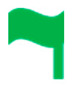	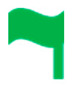	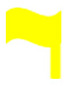	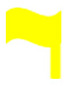	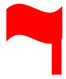	[49]
Lipase from *Aspergillus oryzae* immobilized on SILLP	19′	2-Adamantanone	93	99	56.5	25.5	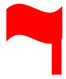	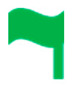	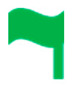	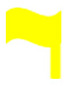	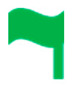	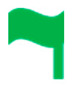	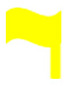	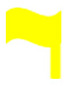	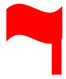	[49]
Lipase from *Candida rugosa* immobilized on SILLP	20′	2-Adamantanone	51	99	56.5	14	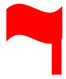	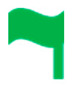	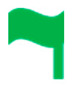	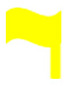	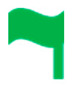	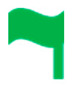	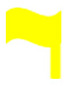	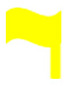	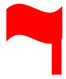	[49]
CALB	21′	Cyclohexanone	92	46	47.1	43.3	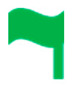	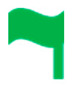	No data	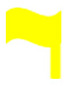	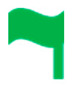	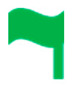	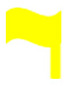	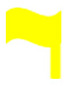	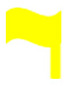	[50]
CALB	22′	Cyclopentanone	95	48	43.8	41.7	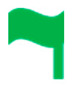	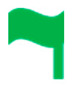	No data	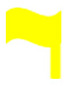	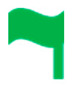	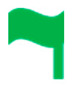	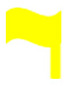	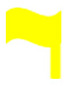	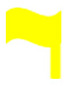	[50]
Ser105Ala	23′	Cyclohexanone	47	97	47.1	22.1	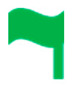	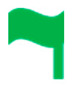	No data	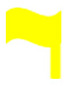	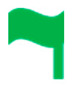	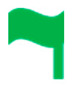	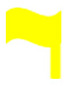	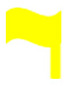	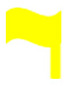	[50]
Ser105Ala	24′	Cyclopentanone	51	99	43.8	22.4	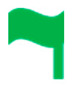	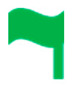	No data	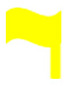	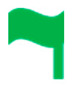	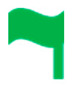	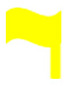	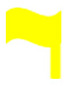	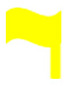	[50]

[^a^] atom economy. [^b^] reaction mass efficiency. [^c^] lipase B from *Candida antarctica.* [^d^] supported ionic liquid-like phase. Flag system: green flag denotes preferred, yellow is acceptable, red is undesirable.

## Data Availability

Data sharing is not applicable for this article.

## Supplementary Materials

The following supporting information can be downloaded at: https://www.mdpi.com/article/10.3390/molecules31132226/s1, File S1: Green Chemistry metrics analysis.

## References

[1] Sartori S.K., Diaz M.A.N., Diaz-Muñoz G. (2021). Lactones: Classification, synthesis, biological activities, and industrial applications. Tetrahedron.

[2] Caprolactone Market Analysis and Forecast. https://researchintelo.com/report/caprolactone-market.

[3] Ntrivala M.A., Pitsavas A.C., Lazaridou K., Baziakou Z., Karavasili D., Papadimitriou M., Ntagkopoulou C., Balla E., Bikiaris D.N. (2025). Polycaprolactone (PCL): The biodegradable polyester shaping the future of materials—A review on synthesis, properties, biodegradation, applications and future perspectives. Eur. Polym. J..

[4] Bińczak J., Dziuba K., Chrobok A. (2021). Recent Developments in Lactone Monomers and Polymer Synthesis and Application. Materials.

[5] Sheldon R.A. (2017). The E factor 25 years on: The rise of green chemistry and sustainability. Green Chem..

[6] Ratti R. (2020). Industrial applications of green chemistry: Status, challenges and prospects. SN Appl. Sci..

[7] Dai C., Zhang J., Huang C., Lei Z. (2017). Ionic liquids in selective oxidation: Catalysts and solvents. Chem. Rev..

[8] Betz D., Altmann P., Cokoja M., Herrmann W.A., Kühn F.E. (2011). Recent advances in oxidation catalysis using ionic liquids as solvents. Coord. Chem. Rev..

[9] Ma Q., Xue Y., Guo J., Peng X. (2023). The Baeyer–Villiger oxidation of cycloketones using hydrogen peroxide as an oxidant. Catalysts.

[10] Vekariya R.L. (2017). A review of ionic liquids: Applications towards catalytic organic transformations. J. Mol. Liq..

[11] Chrobok A. (2010). Baeyer–Villiger oxidation of ketones in ionic liquids using molecular oxygen in the presence of benzaldehyde. Tetrahedron.

[12] Baj S., Chrobok A., Słupska R. (2009). The Baeyer–Villiger oxidation of ketones with bis(trimethylsilyl) peroxide in the presence of ionic liquids as the solvent and catalyst. Green Chem..

[13] Champagne E., Strandman S., Zhu X.X. (2016). Recent developments and optimization of lipase-catalyzed lactone formation and ring-opening polymerization. Macromol. Rapid Commun..

[14] Schrittwieser J.H., Velikogne S., Hall M., Kroutil W. (2018). Artificial biocatalytic linear cascades for preparation of organic molecules. Chem. Rev..

[15] Hollmann F., Kara S., Opperman D.J., Wang Y. (2018). Biocatalytic synthesis of lactones and lactams. Chem. Asian J..

[16] Fürst M.J.L.J., Gran-Scheuch A., Aalbers F.S., Fraaije M.W. (2019). Baeyer–Villiger monooxygenases: Tunable oxidative biocatalysts. ACS Catal..

[17] Ma X.L., Wang Y.H., Shen J.H., Hu Y. (2021). Progress in the synthesis of heterocyclic compounds catalyzed by lipases. Pharm. Front..

[18] de Gonzalo G., Paul C.E. (2021). Recent trends in synthetic enzymatic cascades promoted by alcohol dehydrogenases. Curr. Opin. Green Sustain. Chem..

[19] Silva R., Coelho E., Aguiar T.Q., Domingues L. (2021). Microbial biosynthesis of lactones: Gaps and opportunities towards sustainable production. Appl. Sci..

[20] Fatima S., Zahoor A.F., Khan S.G., Naqvi S.A.R., Hussain S.M., Nazeer U., Mansha A., Ahmad H., Chaudhrye A.R., Irfanf A. (2024). Baeyer–Villiger oxidation: A promising tool for the synthesis of natural products: A review. RSC Adv..

[21] Garcia-Verdugo E., Lozano P., Luis S.V., Fehrmann R., Riisager A., Haumann M. (2014). Biocatalytic processes based on supported ionic liquids. Supported Ionic Liquids: Fundamental and Applications.

[22] Moniruzzaman M., Kamiya N., Goto M. (2010). Activation and stabilization of enzymes in ionic liquids. Org. Biomol. Chem..

[23] Wolny A., Chrobok A. (2021). Ionic liquids for development of heterogeneous catalysts based on nanomaterials for biocatalysis. Nanomaterials.

[24] Itoh T. (2017). Ionic Liquids as Tool to Improve Enzymatic Organic Synthesis. Chem. Rev..

[25] Lee S.H., Doan T.T.N., Ha S.H., Chang W.-J., Koo Y.-M. (2007). Influence of ionic liquids as additives on sol–gel immobilized lipase. J. Mol. Catal. B Enzym..

[26] Zou B., Feng T., Yan Y., Liu F. (2022). Magnetic cross-linked enzyme aggregates based on ionic liquid modification as novel immobilized biocatalyst for phytosterol esterification. Catal. Sci. Technol..

[27] Moniruzzaman M., Kamiya N., Nakashima K., Goto M. (2008). Water-in-ionic liquid microemulsions as a new medium for enzymatic reactions. Green Chem..

[28] Lee J.K., Kim M.-J. (2002). Ionic liquid-coated enzyme for biocatalysis in organic solvent. J. Org. Chem..

[29] Walton A.Z., Stewart J.D. (2002). An efficient enzymatic Baeyer–Villiger oxidation by engineered Escherichia coli cells under non-growing conditions. Biotechnol. Prog..

[30] Lee W.H., Park J.B., Park K., Kim M.-D., Seo J.-H. (2007). Enhanced production of ε-caprolactone by overexpression of NADPH-regenerating glucose 6-phosphate dehydrogenase. Appl. Microbiol. Biotechnol..

[31] Doo E.H., Lee W.H., Seo H.S., Seo J.H., Park J.B. (2009). Productivity of cyclohexanone oxidation in recombinant *Corynebacterium glutamicum*. J. Biotechnol..

[32] Carballeira J.D., Álvarez E., Sinisterra J.V. (2004). Biotransformation of cyclohexanone using immobilized *Geotrichum candidum* NCYC49. J. Mol. Catal. B Enzym..

[33] Silva A.L.P., Batista P.K., Filho A.D., do Nascimento Junior C.S., Reboucas J.S., Vale J.A. (2017). Rapid conversion of cyclohexanone to ε-caprolactone using *Geotrichum candidum*. Biocatal. Biotransform..

[34] Siitonen V., Probst A., Tóth G., Kourist R., Schroda M., Kosourov S., Allahverdiyeva Y. (2023). Engineered green alga *Chlamydomonas reinhardtii* as whole-cell biocatalyst. Green Chem..

[35] Ríos M.Y., Salazar E., Olivo H.F. (2007). Baeyer–Villiger oxidation via lipase-mediated perhydrolysis. Green Chem..

[36] Chávez G., Hatti-Kaul R., Sheldon R.A., Mamo G. (2013). Baeyer–Villiger oxidation with peracid generated In Situ by CaLB-CLEA catalyzed perhydrolysis. J. Mol. Catal. B Enzym..

[37] Drożdż A., Chrobok A., Baj S., Szymańska K., Mrowiec-Białoń J., Jarzębski A.B. (2013). The chemo-enzymatic Baeyer–Villiger oxidation of cyclic ketones with an efficient silica-supported lipase as a biocatalyst. Appl. Catal. A Gen..

[38] Zhang Y., Jiang W., Lv K., Gao X., Zhao Q., Ren W., Wang F., Liu J. (2019). Optimization of chemoenzymatic Baeyer–Villiger oxidation of cyclohexanone to ε-caprolactone using response surface methodology. Biotechnol. Prog..

[39] Drożdż A., Erfurt K., Bielasa R., Chrobok A. (2015). Chemo-enzymatic Baeyer–Villiger oxidation in the presence of Candida antarctica lipase B and ionic liquids. New J. Chem..

[40] Szelwicka A., Zawadzki P., Sitko M., Boncel S., Czardybon W., Chrobok A. (2019). Continuous flow chemo-enzymatic Baeyer–Villiger oxidation with superactive and extra-stable enzyme/carbon nanotube catalyst: An efficient upgrade from batch to flow. Org. Process Res. Dev..

[41] Drożdż A., Chrobok A. (2016). Chemo-enzymatic Baeyer–Villiger oxidation of 4-methylcyclohexanone via kinetic resolution of racemic carboxylic acids: Direct access to enantioenriched lactone. Chem. Commun..

[42] Staudt S., Bornscheuer U.T., Menyes U., Hummel W., Gröger H. (2013). Direct biocatalytic one-pot transformation of cyclohexanol with molecular oxygen into ε-caprolactone. Enzym. Microb. Technol..

[43] Srinivasamurthy V.S.T., Böttcher D., Engel J., Kara S., Bornscheuer U.T. (2020). A whole-cell process for the production of ε-caprolactone in aqueous media. Process Biochem..

[44] Bernhard M.L.J., Gröger H. (2024). Overcoming a conceptual limitation of industrial ε-caprolactone production via chemoenzymatic synthesis in organic medium. ChemSusChem.

[45] Bornadel A., Hatti-Kaul R., Hollmann F., Kara S. (2015). A bi-enzymatic convergent cascade for ε-caprolactone synthesis employing 1,6-hexanediol as a “double-smart cosubstrate”. ChemSusChem.

[46] Irwin A.J., Jones J.B. (1977). Regiospecific and enantioselective horse liver alcohol dehydrogenase catalyzed oxidations of some hydroxycyclopentanes. J. Am. Chem. Soc..

[47] Kotlewska A.J., van Rantwijk F., Sheldon R.A., Arends I.W.C.E. (2011). Epoxidation and Baeyer–Villiger oxidation using hydrogen peroxide and a lipase dissolved in ionic liquids. Green. Chem..

[48] Rios M.Y., Salazar E., Olivo H.F. (2008). Chemo-enzymatic Baeyer–Villiger oxidation of cyclopentanone and substituted cyclopentanones. J. Mol. Catal. B Enzym..

[49] Szelwicka A., Wolny A., Grymel M., Jurczyk S., Boncel S., Chrobok A. (2021). Chemo-enzymatic Baeyer–Villiger oxidation facilitated with lipases immobilized in the supported ionic liquid phase. Materials.

[50] Mazur M., Janeczko T., Gładkowski W. (2022). Lipase-mediated Baeyer–Villiger oxidation of benzylcyclopentanones in ester solvents and deep eutectic solvents. Sci. Rep..

[51] Wang X.P., Zhou P.F., Li Z.G., Yang B., Hollmann F., Wang Y.H. (2017). Engineering a lipase B from Candida antarctica with efficient perhydrolysis performance by eliminating its hydrolase activity. Sci. Rep..

[52] McElroy C.R., Constantinou A., Jones L.C., Summerton L., Clark J.H. (2015). Towards a holistic approach to metrics for the 21st century pharmaceutical industry. Green Chem..

[53] Sheldon R.A. (2021). Biocatalysis in ionic liquids: State-of-the-union. Green Chem..

[54] Guncheva M., Yakimova B. (2025). Diversity of Potential (Bio)Technological Applications of Amino Acid-Based Ionic Liquids. Appl. Sci..

